# Brazilian Green Propolis Inhibits Ox-LDL-Stimulated Oxidative Stress in Human Umbilical Vein Endothelial Cells Partly through PI3K/Akt/mTOR-Mediated Nrf2/HO-1 Pathway

**DOI:** 10.1155/2019/5789574

**Published:** 2019-07-07

**Authors:** Wenwen Yuan, Huasong Chang, Xinying Liu, Shiqiang Wang, Hui Liu, Hongzhuan Xuan

**Affiliations:** ^1^School of Life Science, Liaocheng University, Liaocheng 252059, China; ^2^Center of Bee Industry on Seed-Breeding and Popularization in Shandong Province, Tai'an, China

## Abstract

Propolis has been widely used as a dietary supplement for its health benefits, including cardiovascular protective effects. The aim of this study was to investigate the cytoprotective effects of Brazilian green propolis (BP) against oxidized low-density lipoprotein (Ox-LDL) induced human umbilical vein endothelial cells (HUVECs) damage. Our results suggested that treatment with BP rescued Ox-LDL-stimulated HUVECs cell viability losses, which might be associated with its inhibitive effects on the cell apoptosis and autophagy. We also noticed that BP restored Ox-LDL-stimulated HUVECs oxidative stress, by induced antioxidant gene expressions, including Heme oxygenase-1 and its upstream mediator, Nrf2, which were mediated by the activation of the phosphorylation of PI3K/Akt/mTOR. Pretreatment with wortmannin, PI3K/AKT inhibitor, abolished BP induced Nrf2 nuclear translocation and HO-1 level. Our results demonstrated that BP protected HUVECs against oxidative damage partly via PI3K/Akt/mTOR-mediated Nrf/HO-1 pathway, which might be applied into preventing Ox-LDL mediated cardiovascular diseases.

## 1. Introduction

Atherosclerosis is a complex chronic inflammatory and metabolic disease, which is a consequence of oxidative stress, where homeostasis between endogenous antioxidants and reactive oxygen species is disrupted leading to lipid and protein oxidation in the vascular wall. Accumulation of lipoproteins in the vessel wall provides the initial trigger for vascular inflammation, causing endothelial dysfunction and monocyte recruitment [1]. Oxidized low-density lipoprotein (Ox-LDL) is a crucial factor in triggering the development of atherosclerosis [2]. It not only directly targets vascular endothelial cells (VECs) to induce endothelium injury or dysfunction, but can activate monocytes and macrophages by binding to scavenger receptors leading to the formation of plaque and secretion of proinflammatory cytokines [3, 4]. A mounting evidence indicated that endothelial cells dysfunctions may be harmful to the plaque vulnerability [5, 6]. Thus, protecting the endothelial cells against damage or death has been considered a novel target for the atherosclerotic treatment.

Previous reports suggested that oxidative stress plays an important role in Ox-LDL induced cell damage [7]. The imbalance of redox status of a cell can cause damage to cellular components or induce cell necrosis or apoptosis, and suppressing oxidative stress has the potential to be useful in cardiovascular diseases treatment [8]. Nuclear factor erythroid 2-related factor 2 (Nrf2) is known as key modulator by upregulating of a number of detoxifying antioxidant enzymes, including heme-oxygenase-1 (HO-1), quinone oxidoreductase (NQO-1), glutathione S-transferase (GST), NAD(P)H, and *γ*-glutamyl cysteine synthetase catalytic subunit (GCLC) [9, 10]. Nrf2 is distributed mainly in the cytoplasm and its translocation to the nucleus is inhibited by a Kelch-like ECH-associated protein 1 (Keap1) under normal physiological conditions. Confirmation of Keap1 changes through interacting with other inducers results in the release of Nrf2, which is then free to translocate into the nucleus to regulate basal antioxidant-response elements (ARE) expression including HO-1[11]. Nrf2/HO-1 axis provides a theoretical basis for the therapeutic effects against various oxidative stress-relevant diseases, through counteracting oxidative stress injury to resist inflammation, oxidation, mitochondrial damage, apoptosis, pyroptosis, and autophagy [12]. Additionally, various signaling cascades including mitogen-activated protein kinase (MAPK), phosphatidylinositol 3-kinase (PI3K/Akt), protein kinase C (PKC), c-jun N-terminal kinase (JNK), and extracellular signal-regulated kinase (ERK) participate in Nrf2 activation [13], etc.

Propolis, a resinous material that honeybees (*Apis mellifera* L.) collect from various plant resources, is used widely as a health and functional food [14]. Abundant polyphenolic constituents have been identified in propolis [15]. In recent years phenolic compounds have been considered as intracellular direct antioxidants, by scavenging oxidizing species, and to exert an indirect effect, by inducing the upregulation of the synthesis of endogenous antioxidant enzymes such as superoxide dismutase, catalases, and peroxidases [16]. Our recent studies have demonstrated that propolis has shown excellent antioxidant activities, which might be attributed to the activation of PI3K/Akt/mTOR, an important signaling pathway in mediating cellular survival, proliferation, differentiation, apoptosis, and metastasis [17]. However, the relationship between Nrf2/HO-1 activation and PI3K/Akt/mTOR signal pathway on protecting endothelial cells has not been fully elucidated. The present study was designed to verify the hypothesis that Brazilian green propolis could inhibit apoptosis and autophagy induced by Ox-LDL in HUVECs by PI3K/Akt/mTOR-mediated Nrf2/HO-1 pathway.

## 2. Materials and Methods

### 2.1. Materials

Dulbecco's modified Eagle's medium (DMEM) and fetal bovine serum (FBS) were obtained from Gibco (USA). Sulforhodamine B (SRB), acridine orange (AO), Hoechst 33258, 2′,7′-dichlorodihydrofluorescin (DCHF), and JC-1 were from Sigma Co. (USA). Primary antibodies against *β*-actin, PI3K, and p-PI3K were from Santa Cruz Biotechnology (USA). Primary antibodies against caspase 3, PARP, LC3B, p70S6K, p-p70S6K, p-mTOR, mTOR, Akt, p-Akt, Beclin 1, Atg7, and Nrf2 and secondary antibody (horseradish peroxidase) were from Cell Signaling Technology (USA). Primary antibodies against Bcl-2 and Bax were from ABclonal biotech (USA). Primary antibody against p62 was from BD Transduction Laboratories (USA). Primary antibody against HO-1 was from Abcam (USA). Primary antibody Lamin B was from Beyotime (China). Secondary antibody for immunofluorescence, donkey anti-rabbit IgG Alexa Fluor-488, was purchased from Life Technologies (USA). Wortmannin was obtained from Selleck (USA). Ox-LDL was purchased from Beijing Xiesheng Biotechnology (China). DPPH, ABTS, *α*-tocopherol (Vitamin E), gallic acid, quercetin, and the standards used in HPLC analysis were purchased from Sigma (St. Louis, MO, USA). All other reagents were of ultrapure grade.

### 2.2. Preparation of Ethanol Extracted Brazilian Propolis

Brazilian green propolis collected in Minas Gerais State of Brazil was used in present study, where* Baccharis dracunculifolia* DC was the main botanical source. The extraction method was as follows. 95% (v/v) ethanol was used to extract raw Brazilian green propolis in the dark for 24 h with a constant stirring. The supernatant was filtered to remove the residues. Then the solvent was evaporated in a rotary evaporator under a reduced pressure at 50°C until reaching a constant weight. The ethanol extracted Brazilian green propolis (EEBP) was redissolved in ethanol when it is used. The major chemical constituents were analyzed by 6510 Liquid chromatography-tandem quadrupole time of flight mass spectrometry (HPLC-DAD/Q-TOF-MS). In brief, Proshell 120SB-C18 column (2.1 mm×100 mm, 2.7 *μ*m) was used in the chromatographic experiments. The mobile phases used were methanol/water in gradient elution mode (0~2 min,15%~30%; 10~25 min, 30%~90%; 25~30 min, 90%; 30~31 min, 15%). The sample injection volume was 2 *μ*L, and the concentration of propolis was 10 mg/mL. The column temperature was maintained at 30°C and the flow rate was set to 0.2 mL/min. The UV detector was set at 254 nm. The optimal MS conditions were set as follows: electrospray ionization (ESI); flow rate and temperature were 11.0 L/min and 350°C; nebulizer, 40 psi, respectively.

### 2.3. Determination of Total Phenolic Content (TPC)

The TPC was measured by the modified Folin-Ciocalten method [18]. In brief, 100 *μ*L EEBP (300, 500, and 700 *μ*g/mL) was mixed with 100 *μ*L Folin-Ciocalten reagent and kept for 5 min after vortex in the dark. Then the sample was incubated with 300 *μ*L 2% sodium carbonate at room temperature for 2 h. Finally, 200 *μ*L solution was injected into 96-well plate and measured at 765 nm. In the experiment, different concentrations of gallic acid (0, 5, 10, 20, 40, 60, 80, and 100 *μ*g/mL) were used as a standard and the results were expressed as gallic acid equivalent (GAE) per gram sample.

### 2.4. Total Flavonoids Contents (TFC)

The TFC was determined by the modified method [18]. 150 *μ*L EEBP (300, 500, and 700 *μ*g/mL) was mixed with 10 *μ*L aluminium nitrate (100 g/L) and 10 *μ*L potassium acetate (9.8 g/L). The absorbance was measured at 415 nm after being incubated at room temperature for 1 h. In the experiment, concentrations of quercetin (4-80 *μ*g/mL) were used as a standard to determine TFC in EEBP. The results were expressed as milligrams quercetin equivalent per gram sample.

### 2.5. DPPH Radical-Scavenging Test

DPPH radical-scavenging activity of EEBP was tested by the modified method [18, 19]. Different concentrations of EEBP (70-280 *μ*g/mL) were prepared for the reaction. The reaction mixtures in the 96-well plates consisted of the sample (50 *μ*L) and DPPH radicals (50 *μ*l, 0.5 mg/mL) dissolved in ethanol and kept in the dark for 30 min. The absorbance was measured at 517 nm against a blank. All determinations were performed in triplicate. The scavenging activity of the samples was expressed as the IC_50_ value, the concentration required to scavenge 50% of DPPH radicals.

### 2.6. ABTS Cation Radical-Scavenging Assay

The ABTS cation radical-scavenging activity assay was carried out by ABTS cation radical decolorization with minor modifications [20]. The EEBP solutions (10-220 *μ*g/mL) were prepared as described for the DPPH assay. The ABTS cation radical was prepared by reacting with a 7 mM aqueous solution of ABTS^+^ (7.5 mL) with 140 mM potassium persulphate (132 *μ*L). The mixture was allowed to stand in the dark at room temperature for 16 h before use. Prior to the assays, the ABTS working reagent was diluted with ethanol to give an absorbance of 0.85±05 at 734 nm after equilibration at room temperature. The reaction mixtures in the 96-well plates consisted of the sample (50 *μ*L) and the ABTS^+^ ethanol working solution (100 *μ*L). The mixture was stirred and allowed and left to stand for 10 min in the dark, after which the absorbance was measured at 734 nm against a blank. All determinations were performed in triplicate. The scavenging ability of the samples was expressed as the IC_50_ value, the effective concentration at which 50% of the ABTS radicals were scavenged.

### 2.7. Cell Culture

HUVECs were generous gift from Atherosclerosis Research Institute of Taishan Medical University of China. HUVECs were cultured in high-glucose DMEM medium supplemented with 10% FBS and 100 U/mL penicillin and 100 *μ*g/mL streptomycin. Cells were grown and maintained in a humidified incubator at 37°C and 5% CO_2_. All studies were performed using 80-90% confluent cells before treatment. To induce vascular endothelial damage, HUVECs were treated with designed concentrations of Ox-LDL (20, 40, and 80 *μ*g/mL).

### 2.8. Cell Viability Assay

Cells were seeded in 96-well plates. After treatment with EEBP (1.25, 2.5, and 5 *μ*g/mL) combined with Ox-LDL (40 *μ*g/mL) for 3, 6, and 12 h, cell viability was measured by SRB assay. Briefly, 100 *μ*L 10% trichloroacetic acid was used to precipitate cells at 4°C for 1 h. After 5 washes in deionized water, the cells were stained with 50 *μ*L of 0.4% (W/V) SRB solution at room temperature for 20 min and washed five times with 1% acetic acid. 100 *μ*L of 10 mM Tris base was used to dissolve the bound dye. The optical densities were measured at the wavelength of 492 nm using microplate spectrophotometer.

The viability (%) was expressed as (OD of treated group/OD of Ox-LDL group) ×100%. The viability of the Ox-LDL group was set at 100%.

### 2.9. Acridine Orange (AO) and Hoechst 33258 Staining

AO and Hoechst 33258 were used to test the morphological changes of nuclei and apoptosis, respectively. At 6 h, cells were stained with AO (5 *μ*g/mL) at room temperature for 5 min or stained with Hoechst 33258 (10 *μ*g/mL) for 15 min. After that, cells were observed under a laser scanning confocal microscopy (Olympus FV1200, Japan) or a TE2000S fluorescence microscope (Nikon, Japan), respectively.

### 2.10. Real-Time PCR Analysis

After treatment with EEBP (1.25, 2.5, and 5 *μ*g/mL) combined with Ox-LDL (40 *μ*g/mL) for 6 h, total RNA from HUVECs was extracted by RNA extraction kit (Carry Helix, China) in accordance with the manufacturer's protocol. The cDNA was synthesized from RNA with PrimeScript RT Kit (TaKaRa, Dalian, China) in accordance with the manufacturer's protocol. Quantitative PCR was performed with SYBR Premix EX Taq (TaKaRa, Dalian, China) using a Real-time PCR Detection System (Agilent StrataGene Mx3000, USA). The expression of the housekeeping gene *β*-actin was used to normalize the expression levels, and the results were expressed as 2^-ΔΔCt^. The primer pairs were the following: HO-1 (sense, 5′-TCTTGGCTGGCTTCCTTACC-3′; antisense, 5′-GGATGTGCTTTTCGTTGGGG-3′), Nrf2 (sense, 5′-CAACTACTCCCAGGTTGCCC-3′; antisense, 5′-AGTGACTGAAACGTAGCCGAA-3′), *β*-actin (sense, 5′-GCCGTTCCGAAAGTTGCCT-3′; antisense, 5′-CGCGGCGATATCATCATCCAT-3′).

### 2.11. Immunofluorescence Assay

Immunofluorescence assay was performed as previous method [21]. In brief, cells were fixed in 4% paraformaldehyde (w/v) at room temperature for 15 min and then blocked with 5% donkey serum (v/v) for 20 min. After adding the primary antibody-LC3B (1:100) and second antibody (1:200) (FITC-IgG), a laser scanning confocal microscope (Olympus FV1200, Japan) was used for fluorescence detection. Analysis used the Image-Pro Plus software (USA). Images are representative of three independent experiments.

### 2.12. Western Blotting Analysis

Western blotting analysis was performed as previous method [21]. After treatment with EEBP (1.25, 2.5, and 5 *μ*g/mL) combined with Ox-LDL (40 *μ*g/mL) for 6 h, cells were washed twice with ice-cold PBS. Total cell lysates and nuclear extracts were prepared by cell lysis buffer and a nuclear extraction kit (Thermo Scientific, USA), respectively. Protein concentration was measured by the Bradford method. Equal amounts of protein were separated by 12-15% SDS-PAGE and then transferred to polyvinylidene difluoride (PVDF) membranes. 5% skim milk was used to block the membranes at room temperature for 1 h. After that, the membranes were probed with primary antibodies (1:1000) at 4°C for 16 h and incubated with second antibodies (1:3000-5000) at 37°C for 1 h after three washes in TBST. The bands were visualized using an enhanced chemiluminescent detection kit (Thermo Electron Corp., USA).

### 2.13. Intracellular Reactive Oxygen Species (ROS) Analysis

To determine ROS production, cells were washed with basal DMEM medium after treatment and then stained with 0.5 ml DCHF at 37°C for 30 min in the dark. After three washes with basal DMEM medium, the fluorescence was monitored with a confocal laser scanning microscope (Olympus FV1200, Japan). The photographs were representative of three independent experiments. Results were shown as the relative fluorescence intensity ratio compared with Ox-LDL group.

### 2.14. Mitochondrial Membrane Potential Measurement

JC-1 probe was used to test mitochondrial membrane potential. In brief, JC-1 probe was added to cells after EEBP treatment at 37°C for 15 min in the incubator, and cells were washed three times with basal DMEM medium. Then the fluorescence was monitored with a confocal laser scanning microscope (Olympus FV1200, Japan). Results were shown by ratio of red to green fluorescence as compared with the Ox-LDL group.

### 2.15. Statistical Analysis

Data are from at least three independent experiments and expressed as means ± S.E.M. Statistical analysis involved the paired Student* t* test and ANOVA with SPSS Ins (PASW Statistics 18). Differences were considered statistically significant at* P* < 0.05.

## 3. Results

### 3.1. Components Identified in EEBP

A total of 7 compounds were identified and quantified in EEBP based on HPLC-DAD/Q-TOF-MS analysis (Table 1). As shown in Table 1,* p*-coumaric acid (11.72 mg/g) and artepillin C (39.80 mg/g) were the dominant ingredients in EEBP.

### 3.2. Total Phenolic Contents (TPC), Total Flavonoid Contents (TFC), and Free Radical-Scavenging Activities of EEBP

The contents of TPC and TFC in EEBP were shown in Table 2. The content of TPC was 111.40 ± 4.78 mg GAE/g, and TFC was 83.20 ± 4.54 mg/g. Some studies with ethanoic extracts of green propolis from the state of São Paulo have reported that the phenolic concentrations range from 49 to 100 mg GAE/g, and the flavonoid content was of 51.9 ± 2.4 mg/g [22].

And the DPPH free racial scavenging activity (IC_50_) of EEBP was 179.11 ± 4.09, and ABTS^+^ (IC_50_) was 72.10 ± 0.40, which was lower than Chinese propolis [23].

### 3.3. EEBP Enhanced the Cell Viability and Inhibited Apoptosis in Ox-LDL-Stimulated HUVECs

**Figure 1:**
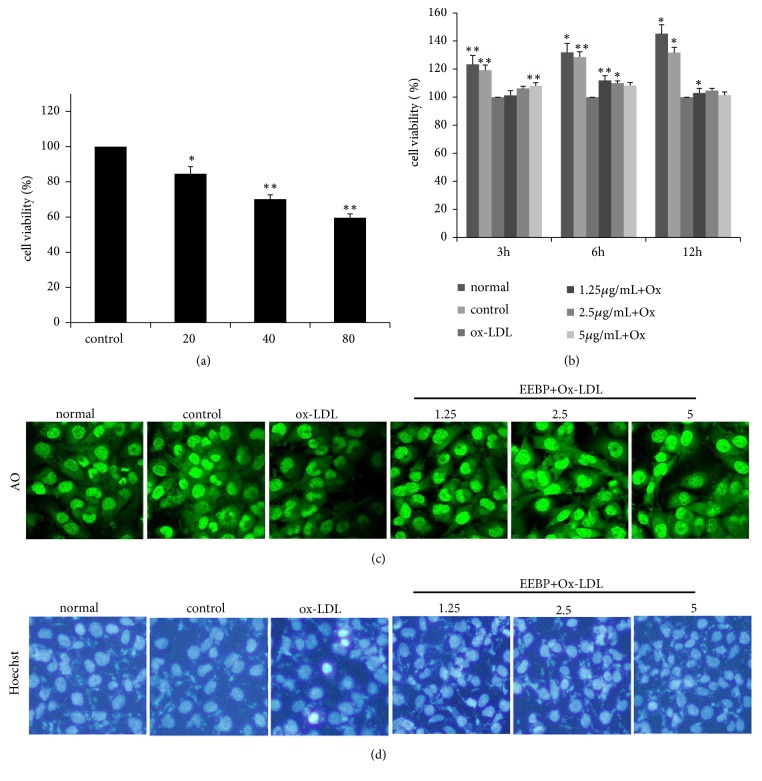
Effects of ethanol extracted Brazilian green propolis (EEBP) on cell viability in Ox-LDL treated HUVECs. (a), Effects of Ox-LDL (20, 40, and 80 *μ*g/mL) on cell viability in Ox-LDL induced HUVECs at 6 h. (b), Effects of EEBP (1.25, 2.5, and 5 *μ*g/mL) on cell viability in Ox-LDL induced HUVECs for 3, 6, and 12 h. Cell viability was tested by SRB assay and illustrated in column figures. (^*∗*^*P* < 0.05, ^*∗∗*^*P* < 0.01 vs Ox-LDL, n=3). Data are means ± S.E.M. (c), AO staining showed treatment with EEBP depressed nuclear condensation and fragmentation in Ox-LDL induced HUVECs (200×). (d), Hoechst 33258 staining suggested that treatment with EEBP inhibited apoptosis in Ox-LDL induced HUVECs (200×).

We firstly observed the effects of different concentrations of Ox-LDL (20, 40, and 80 *μ*g/mL) on HUVECs and found that concentration of Ox-LDL treatment (6 h) within 20-80 *μ*g/mL could inhibit cell survival of HUVECs resulting in decreased cell viability (Figure 1(a)). As a result, in our subsequent studies, we chose Ox-LDL (40 *μ*g/mL) to induce endothelial cells damage. Our previous study showed that poplar propolis could protect HUVECs induced by Ox-LDL, and here we further demonstrated that Brazilian green propolis (1.25, 2.5, and 5 *μ*g/mL) could improve cell viability in Ox-LDL-treated HUVECs at 3, 6, and 12 h. Furthermore, the protective effect of EEBP on Ox-LDL-stimulated HUVECs was more significant at 6 h (Figure 1(b)). AO and Hoechst 33258 staining also indicated that EEBP treatment obviously inhibited nuclear concentration and fragment caused by Ox-LDL (Figures 1(c) and 1(d)).

**Figure 2:**
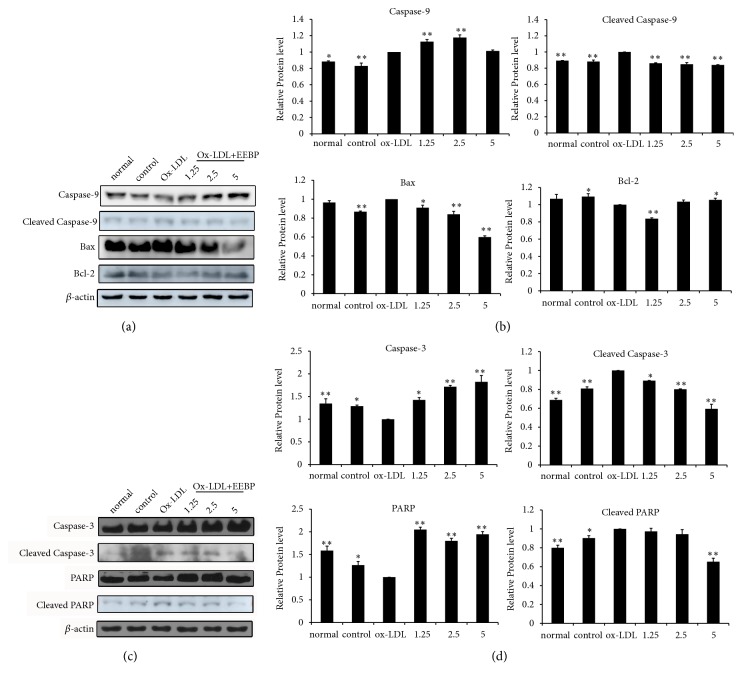
Treatment with EEBP inhibited apoptosis in Ox-LDL-stimulated HUVECs at 6 h. (a), Expression of caspase 9, cleaved caspase 9, Bax, Bcl-2, and *β*-actin in Ox-LDL induced HUVECs. (b), Quantification of relative protein expression quantity in Ox-LDL induced HUVECs at 6 h. (c), Expression of caspase 3, cleaved caspase 3, PARP, cleaved PARP, and *β*-actin in Ox-LDL induced HUVECs. (d), Quantification of relative protein expression quantity in Ox-LDL induced HUVECs at 6 h (^*∗*^*P* < 0.05, ^*∗∗*^*P* < 0.01 vs Ox-LDL, n=3). Data are means ± S.E.M.

To further confirm EEBP inhibited apoptosis in Ox-LDL-stimulated HUVECs, the hallmarks of apoptosis, caspase 9, Bax, Bcl-2, caspase 3, and PARP levels were measured by western blotting analysis. After EEBP treatment, the antiapoptosis protein Bcl-2 level significantly improved, and the level of proapoptosis protein Bax evidently decreased. Furthermore, the cleaved caspase 9, caspase 3, and PARP levels were significantly suppressed after EEBP treatment (Figure 2).

### 3.4. EEBP Suppressed Autophagy in Ox-LDL-Stimulated HUVECs

**Figure 3:**
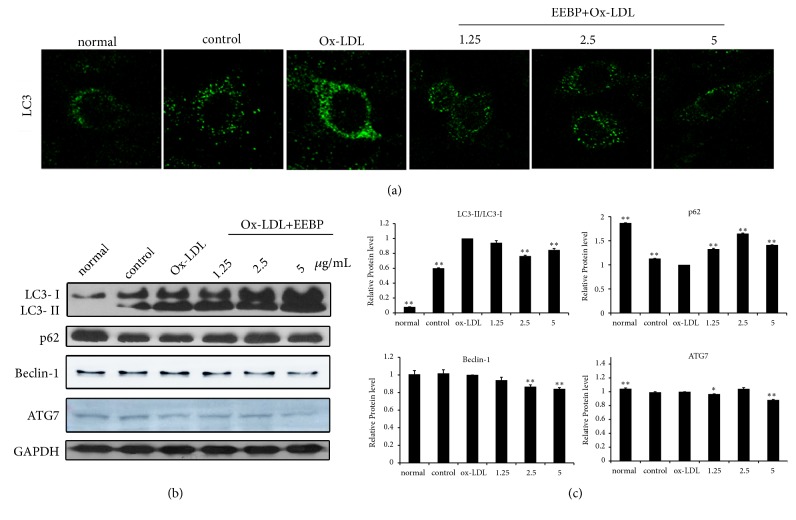
EEBP suppressed autophagy in Ox-LDL-stimulated HUVECs at 6 h. (a), Cells were stained with anti-LC3B antibody for immunostaining. Immunofluorescence graphs showed a decrease of endogenous punctuated LC3 after treatment with EEBP. (b), Expression of LC3B, p62, Beclin 1, and Atg7 in Ox-LDL induced HUVECs. (c), Quantification of relative expression quantity in Ox-LDL induced HUVECs at 6 h, respectively (^*∗*^*P* < 0.05, ^*∗∗*^*P* < 0.01 vs Ox-LDL, n=3). Data are means ± S.E.M.

Besides EEBP suppressed apoptosis, we also wondered about the effect of EEBP on autophagy. As shown in Figure 3(a), when challenged with EEBP (1.25, 2.5, and 5 *μ*g/mL), cells stained with anti-LC3 antibody showed a significantly decreased punctuated pattern compared with Ox-LDL group (Figure 3(a)). Western blotting results also demonstrated that EEBP treatment obviously inhibited LC3 I to LC3 II shift. The levels of p62, the hallmark of autophagic flux, were obviously enhanced after EEBP treatment. Moreover, Beclin 1 and Atg7 levels which also are involved in autophagy also decreased during the process (Figures 3(b) and 3(c)).

### 3.5. EEBP Ameliorated Oxidative Stress in Ox-LDL-Stimulated HUVECs

**Figure 4:**
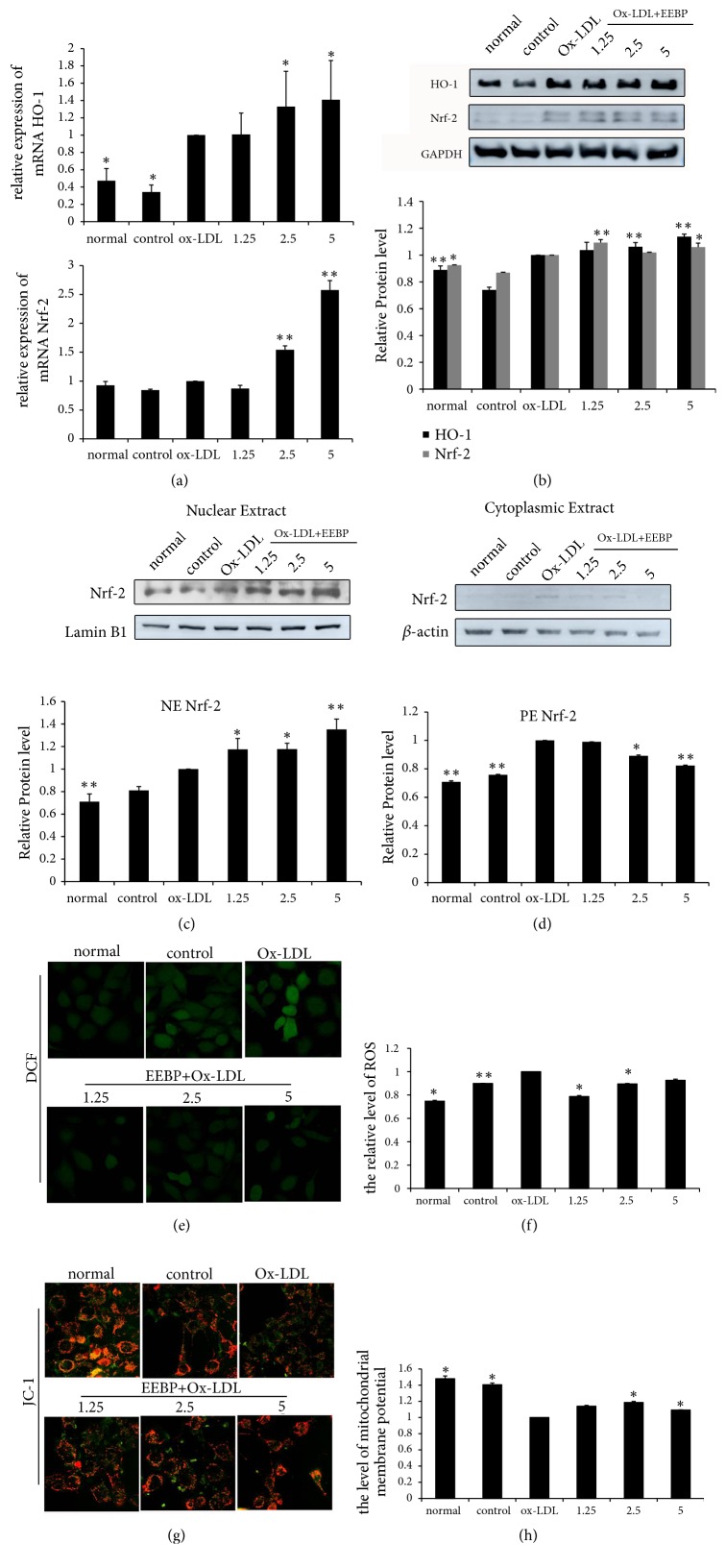
EEBP ameliorated oxidative stress in Ox-LDL-stimulated HUVECs at 6 h. (a), mRNA levels of HO-1 and Nrf2. (b), Expression of HO-1 and Nrf2 of total cell lysate in Ox-LDL-stimulated HUVECs. (c), Expression of Nrf2 of nuclear lysate in Ox-LDL-stimulated HUVECs. (d), Expression of Nrf2 of cytoplasmic lysate in Ox-LDL-stimulated HUVECs. (e), Fluorescent micrographs of ROS obtained in Ox-LDL induced HUVECs at 6 h. (f), Quantification of relative quantity of ROS in Ox-LDL induced HUVECs at 6 h. Values represent the relative fluorescent intensity per cell determined by laser scanning confocal microscopy. (g), Fluorescent micrographs of mitochondrial membrane potential obtained in Ox-LDL induced HUVECs at 6 h. (h), Quantification of relative fluorescent intensity per cell determined by laser scanning confocal microscopy. Values are represented as ratio of red to green fluorescence (^*∗*^*P* < 0.05, ^*∗∗*^*P* < 0.01 vs Ox-LDL, n=3). Data are means ± S.E.M.

The Ox-LDL led to oxidative stress-induced HUVECs damage, which was regarded as an important step in the process of atherosclerosis. To find the protective mechanism of EEBP on Ox-LDL-stimulated HUVECs, we firstly tested the antioxidant genes levels of Nrf2 and HO-1. Nrf2 is a key regulator in the cellular adaptive response to oxidative stress [24]. The activation of Nrf2 is involved in the protection of cells against oxidative stress. In Ox-LDL-stimulated HUVECs, we found that EEBP (2.5 and 5 *μ*g/mL) could dramatically augment the mRAN expression of Nrf2 and its downstream signal molecular –HO-1 at 6 h (Figure 4(a)). Western blotting results also demonstrated that EEBP treatment (2.5 and 5 *μ*g/mL) significantly enhanced HO-1 level (Figure 4(b)). Furthermore, nuclear fractions of EEBP treated cells presented a gradual increase in Nrf2 levels (Figures 4(b)–4(d)). Besides, EEBP treatment significantly inhibited ROS accumulation and elevated mitochondrial membrane potential in Ox-LDL-stimulated HUVECs (Figures 4(e)–4(h)).

### 3.6. EEBP Activated PI3K/Akt/mTOR Signaling Pathway

**Figure 5:**
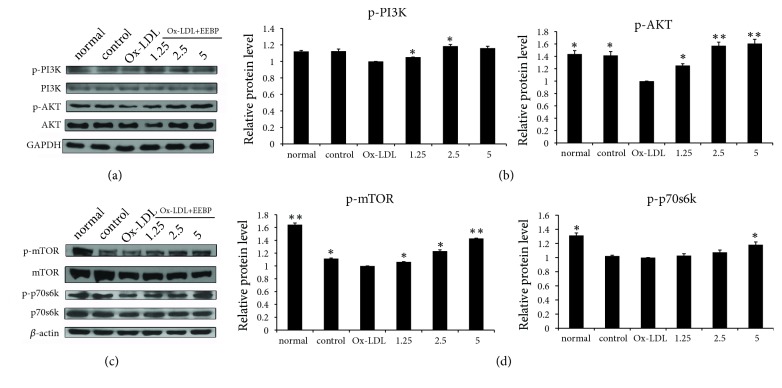
EEBP activated PI3K/Akt/mTOR signaling pathway. (a), Expression of Akt, p-Akt, PI3K, and p-PI3K after treatment with EEBP in Ox-LDL induced HUVECs at 6 h, respectively. (b), Quantification of relative expression quantity of p-Akt and p-PI3K in Ox-LDL induced HUVECs at 6 h, respectively. (c), Expression of mTOR, p-mTOR, p70S6K, and p-p70S6K after treatment with EEBP in Ox-LDL induced HUVECs at 6 h. (d), Quantification of relative expression quantity of p-mTOR and p-p70S6K in Ox-LDL induced HUVECs at 6 h (^*∗*^*P* < 0.05, ^*∗∗*^*P* < 0.01 vs Ox-LDL, n=3). Data are means ± S.E.M.

Furthermore, activation of Nrf2 requires regulation of protein kinases, including the MAPK cascade, PI3K/Akt, and PKC signaling pathways. The phosphorylation of PI3K activated Akt and then mTOR can integrate upstream activating signals through PI3K/Akt pathway and become phosphorylated form, which negatively regulates autophagy [25]. To further determine the antioxidant mechanism of EEBP depressing oxidative stress induced by Ox-LDL, we investigated the effects of EEBP treatment on PI3K/Akt/mTOR signaling pathway. As shown in Figure 5, cells challenged with EEBP evidently increased the phosphorylation levels of PI3K, Akt, and mTOR, and the phosphorylation level of p70S6K, the direct downstream target of mTOR, was also significantly elevated.

### 3.7. EEBP Attenuated Oxidative Stress Was PI3K/AKT/mTOR Dependent

**Figure 6:**
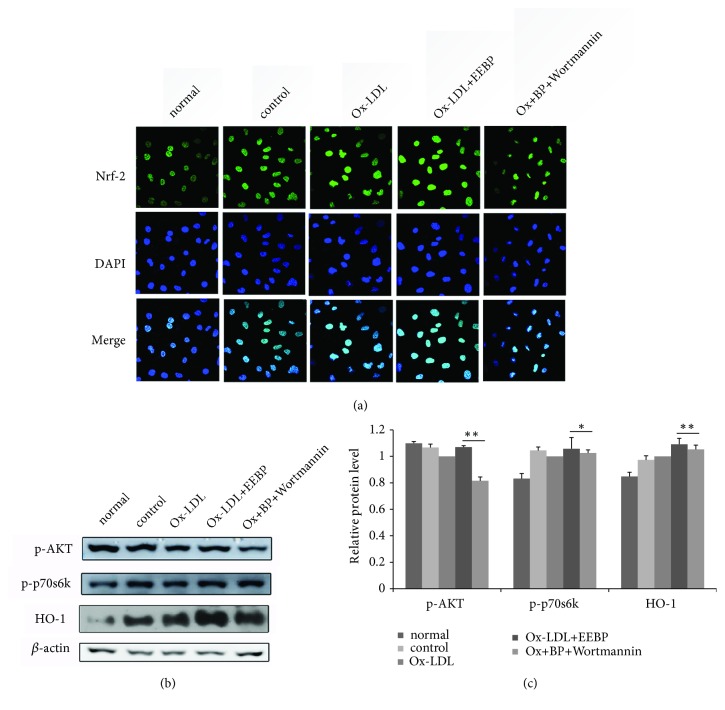
EEBP attenuated oxidative stress was PI3K/AKT/mTOR dependent. (a), Fluorescent micrographs of Nrf2 obtained in Ox-LDL induced HUVECs at 6 h after wortmannin treatment. (b), Expression of p-Akt, p-p70S6K, and HO-1 after wortmannin and EEBP treatment in Ox-LDL induced HUVECs at 6 h, respectively. (c), Quantification of relative expression quantity of p-Akt, p-p70S6K, and HO-1 in Ox-LDL induced HUVECs at 6 h, respectively (^*∗*^*P* < 0.05, ^*∗∗*^*P* < 0.01 vs Ox-LDL, n=3). Data are means ± S.E.M.

Mounting evidence has demonstrated that activation of Nrf2 may be accompanied by Nrf2 nuclear translocation and bind with the ARE sequences. As shown in Figure 6(a), EEBP treatment enhanced Nrf2 accumulation in the nucleus, but Nrf2 level in the nucleus was significantly depressed after wortmannin treatment, a PI3K/Akt inhibitor (Figure 6(a)). Furthermore, the protein expressions of p-Akt, p-p70S6K, and HO-1 were inhibited in cells challenged with wortmannin as well as EEBP treatment (Figures 6(b) and 6(c)), indicating that EEBP attenuated oxidative stress was PI3K/Akt/mTOR dependent.

## 4. Discussion

In this study, we showed the antioxidant activity of the Brazilian green propolis is the key effects for its protecting in vascular endothelial cells against Ox-LDL challenge. Importantly, we have shown that Brazilian green propolis promoted activation of PI3K/Akt/mTOR pathway, further enhancing the cellular antioxidant system. This increased antioxidant activity attenuated apoptosis and inhibited autophagy of HUVECs induced by Ox-LDL.

The chemical constituents of propolis including poplar propolis and green propolis vary greatly according to plant origins and geographical and climatic features of the collection site [26, 27]. Thus we tested the chemical constituents of this sample, and the predominating components in the sample used are* p*-coumaric acid and artepillin C, which can be seen as the markers of green propolis [27]. Moreover, Brazilian green propolis is rich in antioxidant polyphenols, which exerts excellent antioxidant activity.

Oxidative stress is an imbalance between prooxidants and antioxidants, and it has been postulated to be closely associated with cardiovascular diseases, including atherosclerosis. Nrf2 nuclear translocation augments expression of antioxidant-response element HO-1 and NQO1. HO-1 is induced by a variety of conditions associated with oxidative stress. Ishikawa et al. (2001) demonstrated that mildly oxidized LDL markedly induced HO-1 in human aortic endothelial damage [28]. Accumulating evidence demonstrates that natural plant-derived pharmacological modulators upregulate Nrf2/ARE to prevent chronic diseases including cardiovascular disease [29]. Here, BP, one of the plant-derived natural products, shows excellent antioxidant activities through attenuating ROS, protecting mitochondrial membrane potential, activating the antioxidant gene expression of Nrf2 and HO-1. Thus, Brazilian green propolis could improve redox conditions against oxidative stress to protect endothelium cells and further to prevent cardiovascular diseases. Additionally, various signaling cascades including mitogen-activated protein kinase are involved in the activation of Nrf2/HO-1 [30]. The present study showed Brazilian green propolis inhibiting oxidative stress was dependent PI3K/AKT/mTOR activation. Other studies have suggested that poplar type propolis stimulated antioxidant genes expression via p38/Erk-Nrf2 pathway [30]. There are more than 600 kinds of chemical compositions identified in propolis from different plant sources. The chemical compositions of poplar propolis and Brazilian green propolis vary greatly. The chemical constituents of poplar type propolis are mainly flavonoids, phenolic acids, and esters. However,* p*-coumaric acid and artepillin C are the dominant ingredients in Brazilian green propolis. The difference in chemical constituents may lead to the difference in the antioxidant mechanism.

The most inspiring aspect of the present study is that it explores the regulatory mechanism controlling vascular endothelial cells apoptosis and autophagy stimulated by Ox-LDL. Ox-LDL inhibits mTOR and induces apoptosis and autophagy in vascular endothelial cells [31]; Bcl/Bax-caspase9-caspase3 pathway maybe was involved in Ox-LDL-induced HUVECs apoptosis [32]. Many studies also have confirmed that propolis can modulate the expression of the Bcl-2 family genes, producing upregulation of the antiapoptotic Bcl-2 and downregulation of the proapoptotic Bax [33]. Here we found that Ox-LDL-induced HUVECs apoptosis could be inhibited by Brazilian green propolis, in which Bcl2/Bax-caspase9-caspase3 pathway maybe was involved through increasing Bcl-2 and decreasing Bax levels and inhibited the activation of both caspases 9 and 3. On the other hand, autophagy is now known to become dysfunctional in atherosclerosis. Basal autophagy can protect plaque cells against oxidative stress, but excessively stimulated autophagy may lead to endothelium cells death to aggravate plaque rupture. We demonstrated here that the effects of Brazilian green propolis on autophagy might be associated with a mechanistic target of mTOR-dependent signaling pathway.

In summary, propolis alleviated Ox-LDL-induced HUVECs damage by suppressing apoptosis and autophagy. The underlying mechanism may, at least in part, involve activating PI3K/Akt/mTOR and enhancing antioxidant gene HO-1 and Nrf2 expression. Our study provides a potential strategy for prevention and treatment of atherosclerosis and related cardiovascular diseases using natural bee propolis.

## Figures and Tables

**Table 1:** HPLC-DAD/Q-TOF-MS analysis on EEBP.

compounds	Retention time	[M+H]^+^	EEBP (content mg/g)	regression equation
kaempferol	28.4	287.055	1.05	y = 94864x + 129824
caffeic acid	17.2	181.0495	1.51	y = 32392x + 22456
p-coumaric acid	20.22	165.0546	11.72	y = 45243x + 73332
pureonebio	9.8	155.0339	0.34	y = 23525x + 22623
artepillin C	34.7	301.1798	39.80	y = 109286x - 69009
naringenin	27.0	273.0612	0.04	y = 137541x + 229226
ferulic acid	21.4	195.0652	0.14	y = 126715x + 159085

**Table 2:** The contents of TPC and TFC in EEBP and the free radical scavenging activities of EEBP.

sample	TPC (mg GAE/g)	TFC(mg/g)	DPPH(IC_50_)	ABTS^+^ (IC_50_)
EEBP	111.40±4.78	83.20 ± 4.54	179.11 ± 4.09	72.10 ± 0.40

## Data Availability

The data used to support the findings of this study are included within the article.
